# Outcomes following operative vs. non-operative management of blunt traumatic pancreatic injuries: a retrospective multi-institutional study

**DOI:** 10.1186/s41038-016-0065-5

**Published:** 2016-12-09

**Authors:** Poppy Addison, Toni Iurcotta, Leo I. Amodu, Geoffrey Crandall, Meredith Akerman, Daniel Galvin, Annemarie Glazer, Nathan Christopherson, Jose Prince, Matthew Bank, Christopher Sorrentino, Joaquin Cagliani, Jeffrey Nicastro, Gene Coppa, Ernesto P. Molmenti, Horacio L. Rodriguez Rilo

**Affiliations:** 1 Department of Surgery, Hofstra-Northwell School of Medicine, Hempstead, NY USA; 2 Pancreas Disease Center, Northwell Health, Manhasset, NY USA; 3 Department of Biostatistics, Feinstein Institute for Medical Research, Manhasset, NY USA; 4 Northwell Health, 900 Northern Boulevard, Suite 150, Great Neck, NY 11020 USA

**Keywords:** Injury, Management, Non-operative, Operative, Pancreas, Trauma

## Abstract

**Background:**

Traumatic pancreatic injuries are rare, and guidelines specifying management are controversial and difficult to apply in the acute clinical setting. Due to sparse data on these injuries, we carried out a retrospective review to determine outcomes following surgical or non-surgical management of traumatic pancreatic injuries. We hypothesize a higher morbidity and mortality rate in patients treated surgically when compared to patients treated non-surgically.

**Methods:**

We performed a retrospective review of data from four trauma centers in New York from 1990–2014, comparing patients who had blunt traumatic pancreatic injuries who were managed operatively to those managed non-operatively. We compared continuous variables using the Mann-Whitney *U* test and categorical variables using the chi-square and Fisher’s exact tests. Univariate analysis was performed to determine the possible confounding factors associated with mortality in both treatment groups.

**Results:**

Twenty nine patients were managed operatively and 32 non-operatively. There was a significant difference between the operative and non-operative groups in median age (37.0 vs. 16.2 years, *P* = 0.016), grade of pancreatic injury (grade I; 30.8 vs. 85.2%, *P* value for all comparisons <0.0001), median injury severity score (ISS) (16.0 vs. 4.0, *P* = 0.002), blood transfusion (55.2 vs. 15.6%, *P* = 0.0012), other abdominal injuries (79.3 vs. 38.7%, *P* = 0.0014), pelvic fractures (17.2 vs. 0.00%, *P* = 0.020), intensive care unit (ICU) admission (86.2 vs. 50.0%, *P* = 0.003), median length of stay (LOS) (16.0 vs. 4.0 days, *P* <0.0001), and mortality (27.6 vs. 3.1%, *P* = 0.010).

**Conclusions:**

Patients with traumatic pancreatic injuries treated operatively were more severely injured and suffered greater complications than those treated non-operatively. The greater morbidity and mortality associated with these patients warrants further study to determine optimal triage strategies and which subset of patients is likely to benefit from surgery.

## Background

Opinions have differed on modalities of diagnosis and treatment of traumatic pancreatic injuries, since the first documented case nearly 200 years ago [1]. The rarity of the injury, 1–2% of blunt trauma and 5–7% of penetrating [2], makes it difficult for surgeons to develop significant clinical experience in this area. Adding to the complexity, physical exam findings can be absent, laboratory findings non-specific, and imaging unreliable [3]. Once pancreatic injury has been diagnosed, management can be controversial and outcomes difficult to predict.

In order to make the diagnosis, risk factors for injury and confounding factors must be known: demographics, mechanism of injury, presence of other abdominal injuries, time to presentation and utility of imaging modalities. A series from the UK found that the patients suffering pancreatic trauma had a median age of 27 years, the majority were male (M/F = 2.5 to 1), and injured via a blunt mechanism [4]. Pancreatic trauma was first described following a motor vehicle collision [1] and still commonly occurs this way in adults. Sudden blunt force, such as from a steering wheel or bicycle handlebars, compresses the pancreas against the vertebral column; however, any blunt trauma to the abdomen should raise suspicion for pancreatic injury. As the pancreas is situated centrally and retroperitoneally at the level of the L1 and L2 vertebrae, in close proximity to the aorta, inferior vena cava, and portal vein among other viscera [5]; the vast majority of injuries with sufficient energy to injure the pancreas also injure surrounding organs [3]. The classical presentation of upper abdominal pain, leukocytosis, and hyperamylasemia can be absent for the first 24 h following injury [6]. The diagnostic utility of computed tomography (CT) varies significantly with the experience of the radiographic technician and interpreter, with delayed or missed diagnoses occurring between 1.3 and 47% [7]. Considering the lack of clinical experience and pathognomonic findings, diagnosing pancreatic trauma quickly and correctly can be extremely difficult.

Recently, there has been a paradigm shift toward non-operative management of pancreatic injuries in stable patients after blunt trauma [8], particularly in pediatric patients [9], and a growing body of literature comparing outcomes between operative and non-operative management. In a study of pediatric patients, the authors found that both groups of patients had similar lengths of stay (LOS) and readmission rates [9]. There was also a trend toward increased non-pancreatic complications in patients who had pancreatic resections and increased pancreatic complications in the non-operative cohort. In a similar study carried out by Mattix and colleagues regarding outcomes of pediatric patients [10], those treated operatively had higher injury severity scores (ISS). The LOS was longer, and the incidence of pseudocysts, drainage procedures, and pancreatitis was higher in the non-operative group; however, these were not statistically significant [10]. Variables found to be predictive of mortality after pancreatic trauma were increasing age, ISS, hemodynamic compromise, and non-operative management [4].

In a review of 101 patients with blunt pancreatic trauma in 1998, Bradley et al. concluded that without hyperamylasemia, or other reasons for exploratory surgery, the sole deciding factor for management is pancreatic duct involvement [11]. Consistent with his and the findings of others, the Eastern Association for the Surgery of Trauma (EAST) released a level III recommendation regarding the management of pancreatic trauma in 2009 [12]. On the basis of the American Association for the Surgery of Trauma (AAST) grading scheme, injuries that do not involve the duct (grades I and II) warrant drainage, injuries that involve the duct (grade III) warrant resection followed by drainage, and more significant injuries (grades IV and V) had no treatment recommendations made. The common co-existence of traumatic pancreatic injuries with other intra-abdominal injuries makes these guidelines more difficult to follow in actual clinical practice.

We sought to compare outcomes in patients with traumatic pancreatic injuries who were treated operatively to patients who were treated non-operatively. To do this, we performed a retrospective review of patient records from four trauma centers in a large multi-institutional healthcare system in the state of New York. We hypothesize a higher morbidity and mortality rate in patients treated operatively compared to the non-operative group.

## Methods

Patients included in this study were seen at four trauma centers that are part of the Northwell Health System in New York (Cohen Children’s Medical Center (CCMC), Huntington Hospital (HH), Staten Island University Hospital (SIUH), North Shore University Hospital (NSUH)). CCMC and NSUH are designated level I trauma centers, with CCMC specifically being a pediatric level I trauma center. HH is a designated area trauma center, while SIUH is a regional trauma center. All pancreatic injuries were graded using the AAST classification, and were based predominantly on CT imaging. Pancreatic injury grades were changed if intra-operative findings were inconsistent with imaging. Regulatory approval was obtained from the Northwell Health Institutional Review Board (IRB). For the purpose of this study, operative cases were defined as patients requiring any operative intervention. The overwhelming majority of patients had abdominal procedures, and only procedures related to the index traumatic incident and admission were considered. Charts were retrospectively reviewed and the data entered into a Research Electronic Data Capture (REDCap) database. Charts reviewed were paper charts for more recent cases, and some charts had been archived in the form of microfilm.

Descriptive statistics were calculated by group: operative or non-operative subjects (mean ± standard deviation, median, 25th and 75th percentiles for continuous data, and frequencies and percentages for categorical data). The Mann-Whitney *U* test was used to compare operative and non-operative subjects for continuous variables such as age, number of comorbidities, laboratory values, ISS, Glasgow Coma Score (GCS). The chi-square test or Fisher’s exact test as deemed appropriate was used to compare the two groups for categorical variables such as gender, race, and ethnicity.

Time to presentation from injury was analyzed by applying standard methods of survival analysis, i.e., computing the Kaplan-Meier product-limit curves, in which the data was stratified by group. No data were considered censored, and groups were compared using the log-rank test. The median rates for each group were obtained from the Kaplan-Meier/product-limit estimates, and their corresponding 95% confidence intervals were computed using the Greenwood’s formula to calculate the standard error.

Intensive care unit (ICU) LOS and hospital LOS were both analyzed using the above described survival methods; however, the patient was discharged alive from ICU (or hospital), and those subjects who died while in the ICU (or hospital) were considered censored at their date of death. Based on our chart review, no patients were transferred to other institutions. To determine possible confounding factors associated with mortality in the cohort of 61 patients, we performed a univariate analysis comparing the deceased patients to those that were alive, irrespective of whether the patient was treated operatively or non-operatively.

A result was considered statistically significant at the *P* < 0.05 level of significance. All analyses were performed using SAS version 9.4 (SAS Institute Inc., Cary, NC).

## Results

A total of 61 patients were identified, of which 29 were managed operatively and 32 non-operatively. All patients who had penetrating injuries (*n* = 8) were excluded from these analyses.

There was a significant difference between the operative and non-operative groups in median age (37.0 vs. 16.2 years, *P* = 0.016), grade of pancreatic injury (grade I; 30.8 vs. 85.2%, *P* value for all comparisons <0.0001), median ISS (16.0 vs. 4.0, *P* = 0.002), blood transfusion (55.2 vs. 15.6%, *P* = 0.0012), other abdominal injuries (79.3 vs. 38.7%, *P* = 0.0014), pelvic fractures (17.2 vs. 0.00%, *P* = 0.020), ICU admission (86.2 vs. 50.0%, *P* = 0.003), median LOS (16.0 vs. 4.0 days, *P* < 0.0001), and mortality (27.6 vs. 3.1%, *P* = 0.010). The results of our univariate analysis demonstrated that the following factors were associated with mortality irrespective of operative or non-operative treatment; median ISS (25.0 vs. 9.0, *P* = 0.014), blood transfusion within 24 h of admission (88.9 vs. 25.0%, *P* = 0.0005), presence of chest injury (66.7 vs. 26.9%, *P* = 0.05), presence of other abdominal injuries (100 vs. 51.0%, *P* = 0.008), pelvic fractures (33.3 vs. 3.9%, *P* = 0.020), ICU admission (100 vs. 61.5%, *P* = 0.024), post-injury pancreatitis (0.0 vs. 40.0%, *P* = 0.022), low median amylase (33.0 vs. 123.0, *P* = 0.039), and low median serum bicarbonate (18.8 vs. 24.0, *P* = 0.026).

Demographic characteristics are outlined in Table 1, injury and treatment information in Tables 2 and 3, respectively, details of outcomes and complications are found in Table 4, and the results of the univariate analysis to determine factors associated with mortality are found in Table 5.

**Table 1:** Demographic characteristics

Variable	Operative group	Non-operative group	*P* value
*N*	29	32	N/A
Age in years (median)	37.0	16.2	*0.016*
Number of comorbidities (median)	0.00	0.00	0.190
Male sex (%)	65.5	78.1	0.273
Race (%)			
Caucasian	63.0	64.3	
African-American	11.1	7.1	0.940
Ethnicity			
Hispanic	15.8	0.0	
Non-Hispanic	84.2	100.0	0.234

*N/A* not applicable

The italicized values are values that are at or below the statistical level of significance (0.05)

**Table 2:** Details of injury

Variable	Operative group	Non-operative group	*P* value
Type of blunt mechanism (%)			
Fall	17.9	15.6	0.108
MVC	57.1	37.5	
Auto-pedestrian collision	7.1	3.1	
Bicycle accident	3.6	28.1	
Non-accidental trauma	0.0	6.3	
Animal-related	3.6	0.0	
Sports-related	7.1	6.3	
Others	3.6	3.1	
Pancreatic injury grade (%)			
1	30.8	85.2	*<0.0001*
2	34.6	3.7	
3	26.9	0.0	
4	3.9	7.4	
5	3.9	3.7	
Radiologic diagnosis (%)	31.0	90.6	*<0.0001*
Site of injury (%)			
Head	33.3	33.3	0.931
Neck	16.7	9.5	
Body	20.8	28.6	
Tail	29.2	28.6	
Time from injury to presentation (median, h)	0.7	6.8	0.678
Laboratory values (median)			
Lipase	166.0	352.0	0.062
Amylase	89.0	133.5	0.331
Alkaline phosphatase	73.5	134.0	*0.012*
Serum bicarbonate	21.0	25.0	*0.030*
AST	70.0	40.0	0.095
ALT	53.0	35.0	0.101
Ph	7.30	7.32	0.712
HCT	38.0	39.6	0.315
Hb	12.5	13.5	0.734
ISS (median)	16.0	4.0	*0.002*
GCS (median)	15.0	15.0	0.658
Blood transfusion on admission (%)	55.2	15.6	*0.001*
Median blood units transfused	5.5	3.0	0.227
Associated chest injury (%)	44.8	21.9	0.057
Other abdominal injuries (%)	79.3	38.7	*0.001*
Head injury (%)	14.3	9.4	0.700
Spinal fractures (%)	10.7	3.1	0.331
Long bone fractures (%)	21.4	12.5	0.491
Pelvic fractures (%)	17.2	0.0	*0.020*

*ALT* alanine aminotransferase, *AST* aspartate aminotransferase, *ISS* injury severity score, *GCS* Glasgow Coma Score, *MVC* motor vehicle collision

The italicized values are values that are at or below the statistical level of significance (0.05)

**Table 3:** Treatment and operative information

Variable	Operative group	Non-operative group	*P* value
Operative approach (%)			
Open	89.7	N/A	
Minimally invasive	10.3	N/A	
Pancreatic resection (%)			
Yes	46.4	N/A	
No	53.6	N/A	
Type of resection (%)			
Distal pancreatectomy	84.6	N/A	
Other resections	15.4	N/A	
Other procedures^a^			
Drain placement			
Yes	17.2	N/A	
No	82.8	N/A	
Repair of injury			
Yes	13.8	N/A	
No	86.2	N/A	
Evacuation of hematoma			
Yes	13.7	N/A	
No	86.2	N/A	
Negative laparotomy			
Yes	3.5	N/A	
No	96.6	N/A	
Other procedures			
Yes	20.7	N/A	
No	79.3	N/A	
Endoscopic procedure	14.3	9.4	0.695

*N/A* not applicable

^a^Performed in patients who had surgery but did not have pancreatic resections

**Table 4:** Outcomes and complications

Variable	Operative group	Non-operative group	*P* value
Mortality status			
Dead (%)	27.6	3.1	*0.010*
Alive (%)	72.4	96.9	
Cause of death (%)			
CVA	14.3	0.0	
Shock	42.9	0.0	1.000
Others	42.9	100.0	
ICU admission (%)	86.2	50.0	*0.003*
30-day readmission (%)	0.0	12.5	0.116
Surgery 30 days after discharge (%)	0.0	6.3	0.494
Post-injury complications			
Pancreatitis (%)	20.7	46.9	*0.032*
Pancreatic pseudocyst (%)	3.5	6.3	1.000
Pancreatic hematoma (%)	17.2	6.3	0.241
Pancreatic necrosis (%)	13.8	0.0	*0.050*
Endocrine insufficiency (%)	3.5	0.0	0.475
Intra-abdominal fluid collection (%)	69.0	21.9	*0.0002*
TPN requirement (%)	42.9	22.6	0.096
ICU LOS (median, days)	5.0	4.0	0.065
Hospital LOS (median, days)	16.0	4.0	*<0.0001*

*CVA* cerebrovascular accident, *ICU* intensive care unit, *LOS* length of stay, *TPN* total parenteral nutrition

The italicized values are values that are at or below the statistical level of significance (0.05)

**Table 5:** Univariate analysis for independent predictors of mortality

Variable	Dead	Alive	*P* value
Median ISS	25.0	9.0	*0.014*
Blood transfusion (%)	88.9	25.0	*0.0005*
Chest injury (%)	66.7	26.9	*0.050*
Other abdominal injuries (%)	100.0	51.0	*0.008*
Head injury (%)	25.0	9.6	0.232
Spinal fractures (%)	12.5	5.8	0.445
Long bone fractures (%)	25.0	15.4	0.610
Pelvic fractures (%)	33.3	3.9	*0.020*
ICU admission (%)	100.0	61.5	*0.024*
30-day readmission rate (%)	0.0	7.8	1.000
30-day operation post-discharge (%)	0.0	3.9	1.000
Post-injury pancreatitis (%)	0.0	40.4	*0.022*
Post-injury pseudocyst (%)	0.0	5.8	1.000
Post-injury pancreatic hematoma (%)	22.2	9.6	0.273
Post-injury pancreatic necrosis (%)	11.1	5.8	0.481
Endocrine insufficiency (%)	0.0	1.9	1.000
Abdominal fluid collection (%)	77.8	38.5	0.065
TPN requirement (%)	25.8	33.3	1.000
Median laboratory values			
AST	76.0	52.0	0.422
ALT	70.0	37.5	0.492
Lipase	70.0	261.3	0.067
Amylase	33.0	123.0	*0.039*
Bicarbonate	18.8	24.0	*0.026*
Lactate	2.2	3.2	0.376

*ALT* alanine aminotransferase, *AST* aspartate aminotransferase, *ICU* intensive care unit, *ISS* injury severity score, *TPN* total parenteral nutrition

The italicized values are values that are at or below the statistical level of significance (0.05)

## Discussion

We conducted a retrospective review of all patients with blunt traumatic pancreatic injuries seen at four large hospitals over a 24-year time period. Our aim was to compare outcomes in patients with blunt traumatic pancreatic injuries who were treated operatively to those treated non-operatively.

Our results indicate that patients who were treated non-operatively were more likely to be younger and had lower pancreatic injury grades and ISS. Patients treated operatively were older, more severely injured as reflected by higher ISS, and had more associated injuries, notably other non-pancreatic abdominal injuries and pelvic fractures. While there were no differences in initial hemoglobin concentration and hematocrit between groups, more patients who were treated operatively received blood transfusions within 24 h of admission compared to patients treated non-operatively. Patients treated with surgery had longer hospital stays and a higher incidence of pancreatic necrosis, were more likely to develop intra-abdominal fluid collections, and had a higher mortality rate. These occurrences are likely due to the greater severity of injury in this group of patients. However, the non-operative cohort had the only incidents of readmission within 30 days from the date of hospital discharge and a higher percentage of patients with post-injury pancreatitis. The results of univariate analysis demonstrated that ISS, the need for blood transfusion, associated chest, abdominal and pelvic injuries, ICU admission, and low median serum bicarbonate (acidosis) were associated with mortality, independent of operative or non-operative treatment.

Our results imply a higher rate of morbidity and mortality in patients with blunt traumatic injuries of the pancreas who received operative intervention compared to those who did not. This finding, in combination with the results of the univariate analysis, suggests that severity of injuries and not the modality of management are predictive of outcomes. These results call into question the utility of operative intervention in patients with traumatic pancreatic injuries in the context of polytrauma, as well as patients without clear-cut indications for surgery. Indeed, involvement of the pancreas may itself be an indicator of severe abdominal traumatic injury. The need to distinctly identify patients with traumatic pancreatic injuries that stand to benefit from surgery is further underscored by the presence of some patients in the study cohort with grades IV and V injuries that were managed without operation.

In our cohort, we found that grades I, IV, and V injuries tended to be treated non-operatively, while grades II and III were more commonly treated with surgery. While clear-cut indications for surgical exploration such as penetrating abdominal injuries cannot be disputed, the high incidence of associated non-pancreatic abdominal injuries suggests that the decision to operate was not based on the severity of the pancreatic injury alone. In consideration of the high occurrence of multi-organ abdominal injuries, future recommendations for management should examine polytrauma rather than isolated pancreatic injuries in order to be more clinically useful.

The utility of amylase in diagnosing injury to the pancreas, elevation of which indicates pancreatic inflammation and suggests ductal injury, has been controversial. Reports on the application of amylase testing range from no observed correlation between pancreatic trauma and elevations in amylase [13], to affirmation that amylase could indeed signify pancreatic injury [14]. Other reports have shown that contrary to predictions, the increasing severity of pancreatic injury on the basis of presence or absence of ductal injury was not associated with increased serum amylase [15, 16]. Takashima and Matsuno independently found that the amount of time elapsed from the inciting pancreatic trauma to the measurement of amylase may have implications for the diagnostic value of the enzyme in the emergent setting. Serum amylase taken less than 3 [17] or 2 [18] h post-trauma was not diagnostic of blunt pancreatic injury, while delayed measurements were positively correlated with injury. In the present study, mean serum amylase was lower for the operative than for the non-operative group although the difference was not significant. The absence of a statistically significant difference in amylase levels when comparing the two groups of patients in this study could be attributable to a combination of factors: (1) the difference in median time from injury to presentation was not statistically significant, which is crucial considering the change in serum amylase with time; (2) most injuries were grade I and grade II, which by definition are injuries that do not involve ductal disruption. These results complement the current EAST recommendations that amylase levels, although indicative, should not be considered diagnostic for pancreatic trauma [12]. Post-injury pancreatitis and elevated amylase appear to be associated with a lower risk of death according to univariate analysis (Table 5). This association may be explained by the fact that patients with milder injuries present later to hospital, and this leads to higher serum amylase measurements at the time of evaluation. High serum amylase levels are part of the diagnostic criteria for acute pancreatitis.

Patients treated operatively were given blood transfusions more frequently than patients in the non-operative group in spite of similar hemoglobin concentrations and hematocrits at presentation. While blood transfusion does have some detrimental effects, it is prudent to suggest that blood transfusion was given for ongoing hemorrhage in the setting of traumatic injury, or as a result of intra-operative blood loss. It is noteworthy that the amount of units of blood transfused between the groups did not differ significantly. Multiple meta-analyses have demonstrated that each transfused unit of packed red cells increases the risk of infection [19, 20] and is likely due to the immunomodulation that occurs when infusing allogeneic leukocytes [21]. Additionally, transfusion is associated with increased risk of thromboembolism [22], potentially due to increased cell aggregability that occurs during storage [23]. Considering these serious risks, a restrictive transfusion strategy should be considered even for patients managed operatively.

Although our study population was inclusive of all age groups, the higher morbidity and mortality we observed among the operative group might have been mitigated or nullified if pediatric and adult patients had been analyzed separately. In a retrospective review of 167 cases of pediatric blunt trauma to the pancreas leading to grades II and III injuries, Iqbal et al. showed that patients managed operatively by distal pancreatectomy had a quicker return to normal diet (7.8 vs. 2.5 days; *P* = 0.007), fewer complications necessitating further management (including pseudocyst formation) (0 vs. 18%; *P* = 0.001), and shorter time to resolution compared to patients managed non-operatively (22.6 vs. 38.6 days; *P* = 0.05) [24]. The results of Iqbal’s study differ from our findings for several possible reasons: (1) Iqbal’s study examined only patients with grades II and III injuries, while the majority of patients in the non-operative group in our study had grade I injuries; (2) there was no difference in ISS and associated injuries between the patients managed operatively and those managed non-operatively in Iqbal’s study. In summary, patients managed operatively in our cohort may have had worse outcomes than the operatively managed group in Iqbal’s study, due to a higher pancreatic injury grade when compared to patients managed non-operatively, higher ISS, and a higher prevalence of associated injuries. This report also calls to question the extension of the current EAST guidelines to the pediatric population.

All patients who presented to the emergency room with penetrating pancreatic injuries were treated operatively and for that reason were excluded completely from our analyses.

Patients treated by operation were older than patients in the non-operative treatment group. This occurrence might explain the more severe injuries, as older patients might be exposed to higher energy mechanisms of injury such as motor vehicle collisions (MVCs), in comparison to younger patients in the non-operative group who had a larger percentage of bicycle-related injuries. Our completed but unpublished research demonstrates no difference in mortality when comparing pediatric to adult patients with traumatic pancreatic injuries, but comparison of similar age groups or controlling for confounding due to age is needed to draw any definitive conclusions.

Our database, compiled from all blunt traumatic pancreatic injury cases seen at four hospitals from 1990 to 2014, is a relatively large dataset for a very rare injury. In absolute terms, this is a small sample size. While we were able to perform a univariate analyses, the sample size and the small number of patients developing specific outcome measures make it impossible to control confounding using multivariate analysis. A larger sample would have also provided the opportunity to carry out several sub-group analyses.

Despite these limitations, we were able to present a relatively large study on traumatic pancreatic injuries, with regard to the number of patients and variables examined.

Recommendations for future study would be larger scale studies to determine predictors of mortality and survival in patients with isolated traumatic pancreatic injuries, or pancreatic injuries in the setting of polytrauma. Findings from these studies would lay the necessary foundation for the development of consensus guidelines on indications for operative management and procedures of choice in patients with traumatic pancreatic injuries.

## Conclusions

Operative management of traumatic pancreatic injuries in our cohort appears to have been carried out in more severely injured patients who were already at risk of worse outcomes compared to the patients managed non-operatively. The actual effect of modality of management on patient outcomes is difficult to ascertain with a small sample size. While surgery may have been undertaken on account of injury severity and mechanism, further studies need to be carried out to determine the subset of patients who are likely to benefit from surgery and what group of patients may be given a trial of non-operative management. More investigation needs to be carried out on the subject of optimal management of patients with pancreatic injury in the setting of polytrauma.

## Abbreviations

ALT: Alanine aminotransferase
AST: Aspartate aminotransferase
GCS: Glasgow Coma Score
ICU: Intensive care unit
ISS: Injury severity score
LOS: Length of stay
